# Identification of Key Determinants of Cerebral Malaria Development and Inhibition Pathways

**DOI:** 10.1128/mbio.03708-21

**Published:** 2022-01-25

**Authors:** Sung-Jae Cha, Xiang Yu, Brian D. Gregory, Yong Seok Lee, Tomoko Ishino, Robert O. Opoka, Chandy C. John, Marcelo Jacobs-Lorena

**Affiliations:** a Johns Hopkins Bloomberg School of Public Health, Department of Molecular Microbiology and Immunology and Malaria Research Institute, Baltimore, Maryland, USA; b Department of Biology, University of Pennsylvania, Philadelphia, Pennsylvania, USA; c Department of Biology, College of Natural Sciences, Soonchunhyang University, Asan, Chungnam, South Korea; d Division of Molecular Parasitology, Protea-Science Center, Ehime University, Toon, Ehime, Japan; e Department of Paediatrics and Child Health, Makerere University School of Medicine, Kampala, Uganda; f Ryan White Center for Pediatric Infectious Diseases and Global Health, Department of Pediatrics, Indiana University School of Medicine, Indianapolis, Indiana, USA; Washington University School of Medicine

**Keywords:** IGF1, biomarker, cerebral malaria, sporozoite, vascular injury

## Abstract

Cerebral malaria (CM), coma caused by Plasmodium falciparum-infected red blood cells (iRBCs), is the deadliest complication of malaria. The mechanisms that lead to CM development are incompletely understood. Here we report on the identification of activation and inhibition pathways leading to mouse CM with supporting evidence from the analysis of human specimens. We find that CM suppression can be induced by vascular injury when sporozoites exit the circulation to infect the liver and that CM suppression is mediated by the release of soluble factors into the circulation. Among these factors is insulin like growth factor 1 (IGF1), administration of which inhibits CM development in mice.

## INTRODUCTION

Malaria is caused by parasites of the genus *Plasmodium* and is transmitted by *Anopheles* mosquitoes. Rapid expansion of parasite drug resistance, mosquito insecticide resistance and lack of protective vaccines are limiting efforts to eliminate malaria. WHO reported over two hundred million cases and around half million deaths caused by malaria infection in 2019, and 87 countries are still considered to be endemic (1). About 1% of malaria infections develop severe malaria, including cerebral malaria (CM), and the development of CM is much more common in children under 5 years old than in adults in Africa. Of note, the latest World malaria report shows about 67% of malaria deaths occurred in children aged under 5 years (1).

*Plasmodium* sporozoites move from the mosquito bite site to the host liver via the blood circulation and initiate their vertebrate life cycle by infecting hepatocytes, where they differentiate into thousands of merozoites. These are released into the circulation and infect RBCs, inducing disease symptoms. Among all malaria symptoms, CM is one of the most lethals, as it induces an unarousable coma. CM treatment relies mainly on anti-parasite drugs which are not effective to alleviate CM-associated immunopathology. If left untreated, CM is fatal within 24–72 h; early diagnosis and immediate intensive care are crucial for patient survival (2). Unfortunately, 15–20% of CM patients die, even with antimalarial treatment, and up to 24% of the survivors suffer neurological complications and cognitive impairment post recovery (1–3). Since human CM samples are collected from patients presenting symptoms, investigation of human CM development is limited to the final stages of disease. In view of this limitation, the C57/B6 mouse and rodent malaria parasite P. berghei ANKA have been widely investigated. Whereas animal models of CM do not completely recapitulate human CM (4), a number of pathophysiological processes are common, including i) pro-inflammatory spleen immune activation by infected red blood cells (iRBCs), ii) iRBC-mediated brain vascular inflammation, and iii) subsequent blood brain barrier (BBB) disruption (3, 5). Notable, most investigations infect mice via inoculation of iRBCs, bypassing the initial steps of liver infection by sporozoites (3, 4, 6).

We became aware of the importance of the liver stage with the unexpected finding that the mode of sporozoite liver entry profoundly influences CM incidence. Sporozoites move from the mosquito bite site to the liver via the blood circulation and exit in the liver primarily by traversing Kupffer cells, a process that is mediated by the interaction of the sporozoite surface GAPDH ligand with the Kupffer cell CD68 receptor (7, 8). In the absence of the receptor in CD68 knockout (KO) mice, sporozoite liver invasion occurs by breaching the two cell types lining the liver vessels - endothelial and Kupffer, reducing infection is by ∼70% compared with wild type (WT) controls (7). Surprisingly, we found that CM incidence of CD68 KO mice is substantially reduced compared to WT controls when mice are infected with sporozoites but importantly, not when bypassing the liver by infection with iRBCs. Here we report on experiments to investigate the causes of this reduction.

## RESULTS

### Sporozoite infection of CD68 KO mice triggers CM inhibitory pathways.

**FIG 1:**
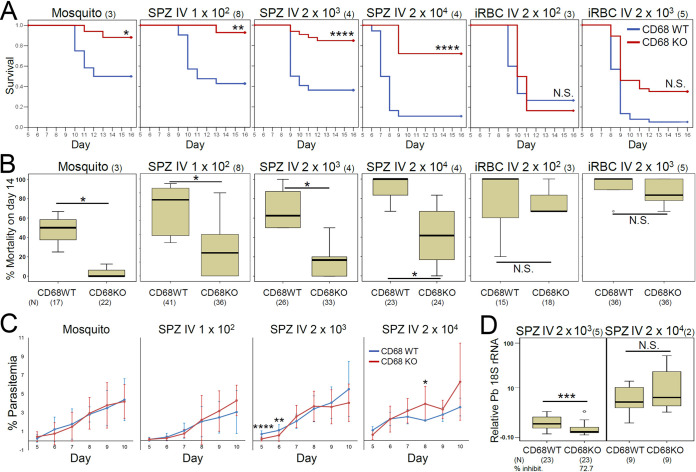
Development of CM is significantly reduced in CD68 KO mice infected with P. berghei sporozoites. (A) Kaplan-Meier survival analysis shows that cumulative survival of sporozoite-infected CD68 KO mice is greater than WT mice when infected with sporozoites, but not when infected with iRBCs. (B) Median mortality on day 14. (C) Parasitemia on day 7 in sporozoite-infected mice. (D) Parasite liver load (‘Relative P. berghei 18S rRNA’) at 42 h postinfection of mice infected with different numbers of sporozoites. (B–D) *P* value was calculated with Mann-Whitney U test. The number in parenthesis next to the parasite dose denotes the number of independent biological repeats, and the number in parenthesis below the mouse genotype denotes total number of mice (*N*) analyzed. Numbers of mice in (A) are the same as in (B). % inhibition was determined using median values in each group. *P* values (* ≤0.05, ** ≤0.01, *** ≤0.001, **** ≤0.0001). N.S., not significant. ‘Mosquito’: mice infected by the bite of one infected mosquito. SPZ IV: infection by intravenous injection of the indicated number of sporozoites. iRBC IV, infection by intravenous injection of the indicated number of infected RBCs.

Most WT mice infected with sporozoites developed CM-associated (CM in short) symptoms such as ruffled fur, hunching, wobbly gait, limb paralysis, convulsions and coma, and mortality quickly increased between 9- and 11-days postinfection (dpi) (Fig. 1A) (9). Surprisingly, CM development and mortality of sporozoite-infected CD68 KO mice was significantly reduced. Importantly, no such differences were observed when the liver stage was bypassed by infection with blood-stage parasites (Fig. 1A and B). Mice showing CM phenotype lost about 10% body weight and their BBB was disrupted while mice showing no CM-associated (NCM in short) phenotypes had no body weight change nor BBB disruption (Fig. S1A, B in the supplemental material). Blood parasitemia of WT and CD68 KO mice is not significantly different in low-dose infections such as mosquito biting or injection of 100 sporozoites. Higher dose infection, with 2,000 or 20,000 sporozoites, showed equivalent parasitemia in CD68 KO and wild-type mice after day 9 (Fig. 1C), arguing against parasitemia being a factor in the difference of CM development. In addition, the CM-resistant phenotype of CD68 KO mice is not due to reduced parasite hepatocyte load because the CM-resistant phenotype of CD68 KO mice is maintained even with similar parasite liver burdens of artificially high-dose infections (2 × 10^4^; Fig. 1D and A).

10.1128/mbio.03708-21.1 FIG S1 CM-associated phenotype development. (A) At day 8–9, mice that developed CM-associated phenotypes had lost significant body weight unlike their infected NCM counterparts. *N*, total number of mice assayed. (B) Evan’s Blue dye penetrates the brain of CM mice but not of their infected NCM counterparts, indicating loss of blood-brain barrier. Box plot shows quartiles, median, and maximum. *P* value was calculated with the Mann-Whitney U test. *P* value (**** ≤0.0001). Download FIG S1, TIF file, 2.1 MB. Copyright © 2022 Cha et al. 2022 Cha et al. https://creativecommons.org/licenses/by/4.0/ This content is distributed under the terms of the Creative Commons Attribution 4.0 International license.

### Soluble CM-inhibitory factors enter the circulation when sporozoites infect CD68 KO mice.

**FIG 2:**
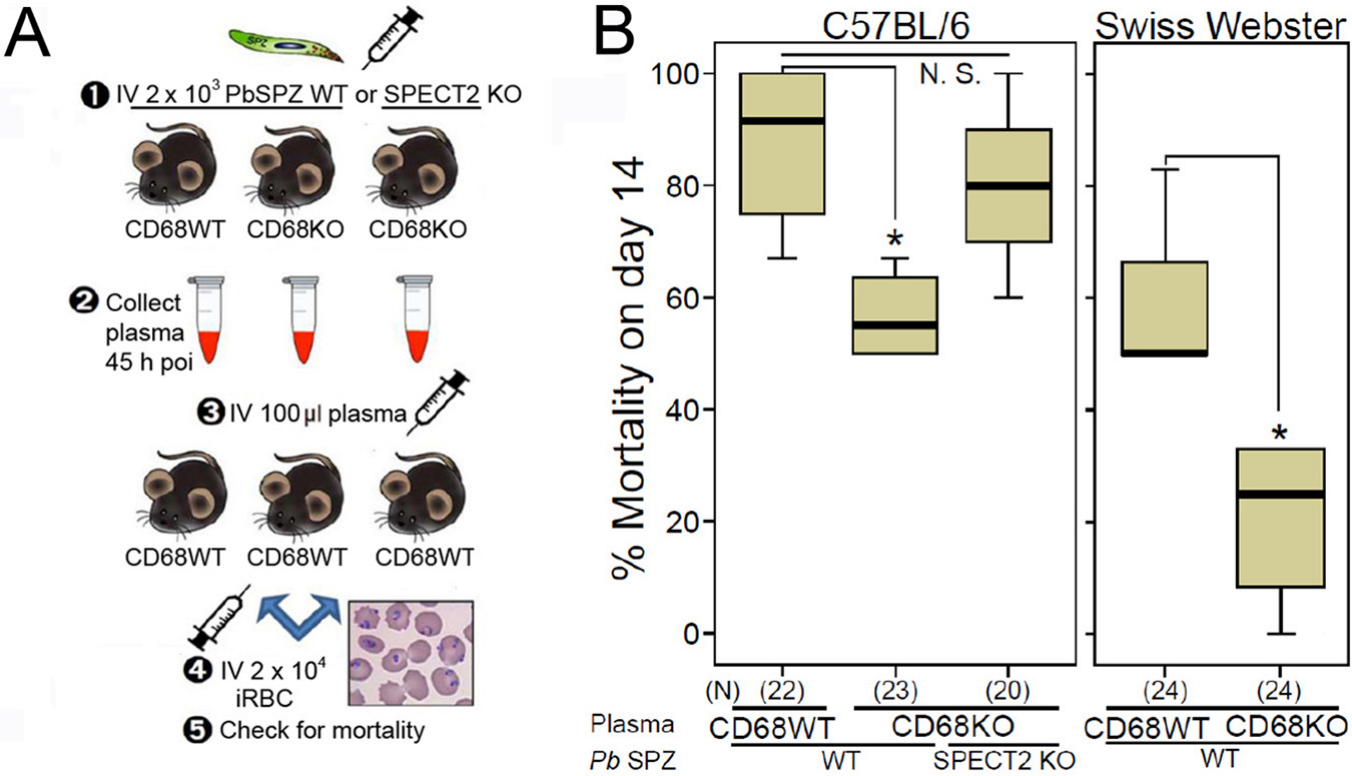
The plasma of sporozoite-infected mice contains soluble factors that modulate CM development. (A) Schematic diagram of the plasma transfer assay. ‘poi’: postinfection. (B) Percent mortality on day 14 poi of mice that received plasma from WT or CD68 KO mice, or CD68 KO mice infected with SPECT2 KO sporozoites. Recipients were inbred wild type C57BL/6 (left panel) or outbred Swiss Webster mice (right panel), as indicated on the top of the panels. Data pooled from four independent experiments. *P* value was calculated with the Mann-Whitney U test. All box plots show quartiles, median, and maximum. The number in parenthesis (N) denotes the total number of mice assayed. *P* values (* ≤0.05). N.S., not significant.

We hypothesized that *Plasmodium* sporozoite liver infection of CD68 KO mice induces the secretion of soluble CM-inhibitory factors. To test this, we collected plasma from sporozoite-infected CD68 KO mice on 2 dpi, when parasites are developing in the liver (9, 10). Plasma collected from sporozoite-infected WT mice served as controls. An additional control consisted of administering mice P. berghei SPECT2 KO sporozoites which lack cell traversal machinery (11). The collected plasma was transferred into WT mice, followed by infection of the recipients with blood-stage parasites (Fig. 2A). As shown in Fig. 2B, CM frequency was significantly lower in mice that received plasma from sporozoite-infected CD68 KO mice (Fig. 2B, left panel; Movie S1 in the supplemental material) compared to mice that received plasma from sporozoite-infected WT mice. This reduction was also observed when the recipients were outbred Swiss Webster mice (Fig. 2B right panel), implying that the CM-inhibitory plasma factors of CD68 KO mice act in a non-strain-specific way. Plasma from SPECT2 KO sporozoite-infected CD68 KO mice had no significant CM inhibitory effect (Fig. 2B left panel), implying that sporozoite cell traversal is key to induce secretion of CM inhibitory plasma factors.

10.1128/mbio.03708-21.15 MOVIE S1 A representative result of plasma transfer assay in Fig 2A. Movie clip shows difference in CM-associated phenotype development between two mice groups on the day 6 after injection of 2 × 10^4^ infected RBC per mouse. As described in Fig 2B, the left cage contains WT C57BL/6 mice that received plasma from WT mice, and the right cage contains WT C57BL/6 mice that received plasma from CD68 KO mice. Plasmas were collected from WT and CD68 KO mice two days after intravenous injection of 2,000 sporozoites. Download Movie S1, MOV file, 10.8 MB. Copyright © 2022 Cha et al. 2022 Cha et al. https://creativecommons.org/licenses/by/4.0/ This content is distributed under the terms of the Creative Commons Attribution 4.0 International license.

### Analysis of soluble plasma factors of CM-negative and CM-positive mice.

**FIG 3:**
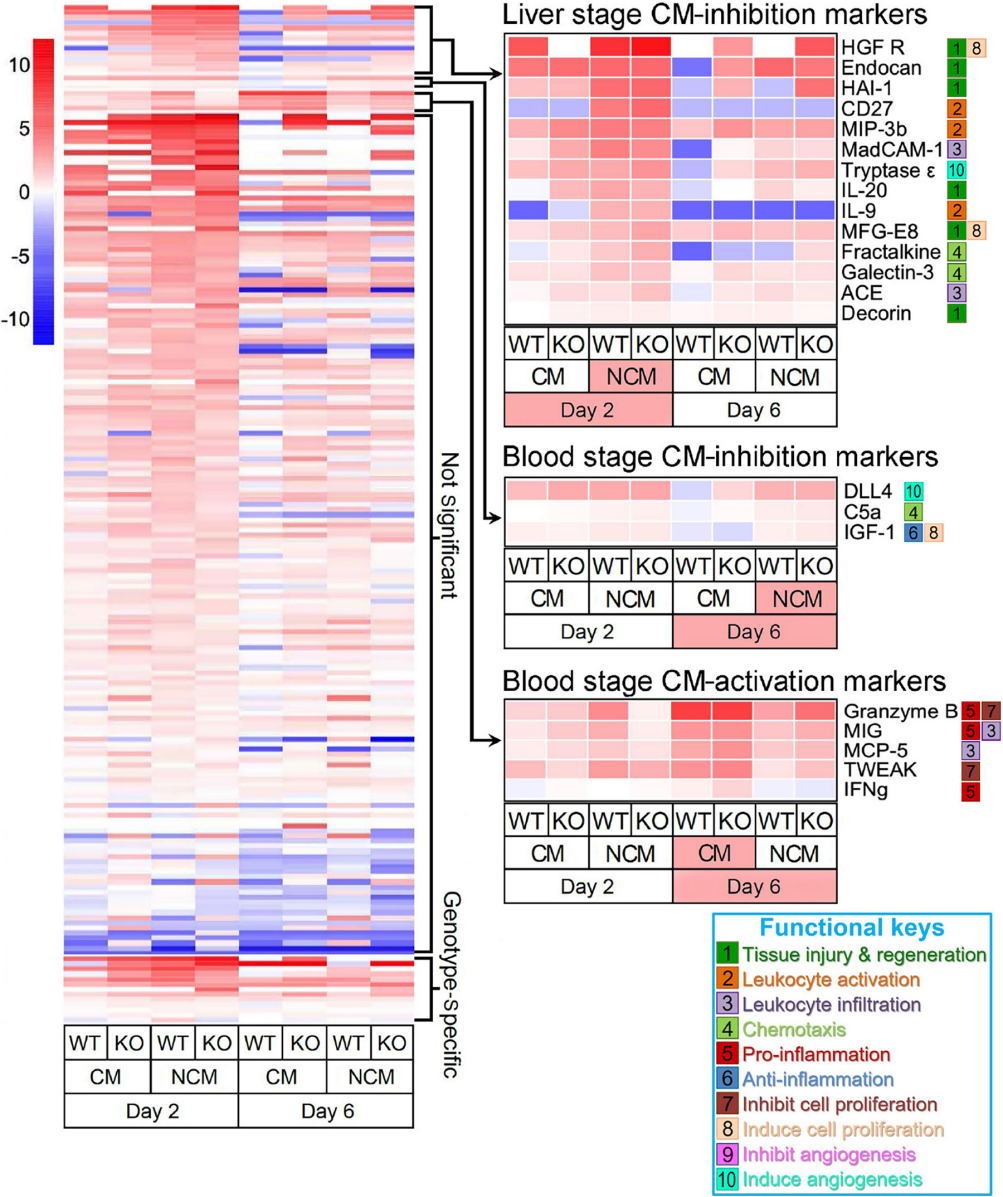
Analysis of the plasma soluble factors that modulate CM development. Heat map of multiplex ELISAs of plasma collected from WT or CD68 KO mice infected with 2,000 sporozoites, or controls injected with mock preparations from non-infected mosquito salivary glands. Color coded fold differences (log_2_) of median relative to WT mock treated controls. Twenty-two out of 200 biomarkers tested showed significant difference (*P < *0.05) between CM and NCM phenotypes, independent of CD68 genotype. These biomarkers were classified into three groups (right panels): Fourteen CM-inhibition markers were upregulated in NCM mice during liver stages (2 dpi); Three CM-inhibition biomarkers were upregulated in NCM mice during blood stages (6 dpi); Five CM-activation biomarkers were upregulated in CM mice during blood stages (6 dpi). 10 functional keys denote the known functions of each biomarker. Data pooled from two or four independent repeats.

To identify soluble factors that are responsible for CM inhibition, we analyzed 200 plasma biomarkers for differences between WT and CD68 KO sporozoite-infected mice using multiplex ELISAs as illustrated in Fig. S2 in the supplemental material. Heat maps and concentration of each biomarker are presented in Fig. 3 and Table S1. Of these, 13 biomarkers (bottom of Fig. 3, left panel; Table S1A, column AI-AM) showed different activation that were genotype-specific but did not correlate with CM phenotype. Importantly, 22 out of 200 biomarkers were predictive of CM phenotype regardless of mouse genotype (WT or CD68 KO). Of these, 14 markers were upregulated at 2 dpi (liver stage) in NCM mouse plasma (Fig. 3, upper right panel, Table S1A, column AT). These biomarkers can be functionally classified as: i) tissue injury and regeneration; ii) leukocyte activation; iii) chemotaxis; iv) leukocyte infiltration; and v) fibrinolysis (Fig. 3; Fig. S3A). Less stringent statistics identified additional liver stage biomarkers showing significant differences between CM and NCM phenotypes (Fig. S4A; Table S1A, columns BD). Liver-stage plasma assays strongly imply that only NCM mice, not CM mice, experience significant tissue injury during liver stages, as NCM plasma displays enhanced expression of Th1 (Galectin-3, ACE, MIP-3b, CD27) and Th2 (IL-9, IL-33, and MMP-10) immune markers (12–18). Tissue injury activates proinflammatory Th1 immunity, followed by regenerative Th2 immune activation that inhibits Th1 inflammation (19).

10.1128/mbio.03708-21.2 FIG S2 Schematic diagram of the plasma assay for Fig. 3 with multiplex ELISA analysis (Quantibody Array, RayBiotech, Inc). Blue numbers (11 to 15) indicate the statistical comparisons made in Fig. S3 and S4, and Table S1. Download FIG S2, JPG file, 0.4 MB. Copyright © 2022 Cha et al. 2022 Cha et al. https://creativecommons.org/licenses/by/4.0/ This content is distributed under the terms of the Creative Commons Attribution 4.0 International license.

10.1128/mbio.03708-21.3 FIG S3 Functional identification of plasma biomarker groups associated with mouse CM development (related to Fig. 3). (A) Fourteen liver stage CM-inhibition biomarkers were classified into five functional groups. (B) Three blood stage CM-inhibition biomarkers were classified into three functional groups. (C) Five blood stage CM-activation biomarkers were classified into three functional groups. (A–C) Blue and red dots represent marker concentrations (pg/ml) in plasma pooled from five CD68 WT or KO mice, respectively. Data pooled from two or four independent repeats. *P* value was calculated with one-way ANOVA TukeyHSD test. *P* values (* ≤0.05, ** ≤0.01, *** ≤0.001, **** ≤0.0001). Download FIG S3, JPG file, 1.1 MB. Copyright © 2022 Cha et al. 2022 Cha et al. https://creativecommons.org/licenses/by/4.0/ This content is distributed under the terms of the Creative Commons Attribution 4.0 International license.

10.1128/mbio.03708-21.4 FIG S4 Functional classification of biomarkers identified with the less stringent ANOVA LSD statistical test (related to Fig. 3). (A) Liver stage CM-inhibition markers can be classified into six different groups based on their attributed functions. (B) The angiogenesis marker Cystatin C was identified as a blood-stage CM-inhibition marker. (C) Three chemotaxis markers were identified as blood-stage CM-activation markers. Blue and red dots represent concentration (pg/ml) of each marker in pooled plasma from five each CD68 WT and KO mice, respectively. *P* value was calculated with one-way ANOVA LSD test. *P* values (* ≤0.05, ** ≤0.01, *** ≤0.001, **** ≤0.0001). Download FIG S4, JPG file, 1.1 MB. Copyright © 2022 Cha et al. 2022 Cha et al. https://creativecommons.org/licenses/by/4.0/ This content is distributed under the terms of the Creative Commons Attribution 4.0 International license.

10.1128/mbio.03708-21.9 TABLE S1 (A) The concentration of 200 plasma biomarkers determined by multiplex ELISA assays. The median, mean, and standard deviation of pg/ml concentration of each group of plasma samples were determined. Plasma biomarkers are identified with Uniprot ID (https://www.uniprot.org) on the left. Individual cases (1∼10) were used for comparison between WT and CD68 KO plasma in the same CM phenotype group. TukeyHSD test identified 13 genotype-specific markers showing significant difference (*P* < 0.05) between genotypes at least in one comparison. The 187 remaining biomarkers were further analyzed with TukeyHSD test to compare between CM and NCM group using combined cases (11 to 15) which pooled WT and CD68 KO cases according to phenotype and collection time (see Fig. S3 and 3). Twenty-two phenotype-specific markers show significant difference (*P* < 0.05) between two CM-associated phenotypes at 2 dpi or 6 dpi (Fig. 3). The 165 remaining biomarkers were further analyzed with LSD test to compare between CM and NCM group using phenotype cases ([Bibr B11][Bibr B12][Bibr B15]) and identified 20 phenotype-specific markers showing significant *P* value (Fig. S4). Only significant *P* values (<0.05) are denoted. Each biomarker reference is noted to the right of the *P* value. (B) The manifestation of human plasma samples for multiplex ELISA assays. Age, sex, CM-related phenotypes, associated sample grouping, and notes of 40 pediatric subjects were described. (C) The concentration of 440 human plasma biomarkers determined by multiplex ELISAs (Fig. 7A). The median, mean, and standard deviation of pg/ml concentration of each group of plasma samples were determined. Plasma biomarker can be identified with Uniprot ID (https://www.uniprot.org) on the left. TukeyHSD test identified 18 biomarkers showing significant difference (*P* < 0.05) between two CM-associated phenotypes. Only significant *P* values (<0.05) were denoted. Each reference for the biomarkers was notified on the right of the *P* value. (D) The concentration of 640 WT mouse plasma biomarkers determined by multiplex ELISA assays 10 days after infection with 2,000 sporozoites (Fig. 7B). The median, mean, and standard deviation of pg/ml concentration of each group of plasma samples were determined. Plasma biomarker can be identified with Uniprot ID (https://www.uniprot.org) on the left. TukeyHSD test identified 12 biomarkers showing significant difference (*P* < 0.05) between two CM-associated phenotypes. Only significant *P* values (<0.05) were denoted. Each reference for the biomarkers was notified on the right of the *P* value. (E) The concentration of 224 human and mouse plasma biomarkers determined by multiplex ELISAs (Fig. 7C). The median, mean, and standard deviation of pg/ml concentration of each group of plasma samples were determined. Plasma biomarker can be identified with Uniprot ID (https://www.uniprot.org) on the left. After exclusion of biomarkers showing significant difference between human and mouse with two-way ANOVA test, six common biomarkers showing similar trends between human and mouse with more than 1.5-fold difference between two CM-related phenotypes were identified. Each reference for the six biomarkers was notified on the right. (F) Biomarker activation profiles in four significantly conserved pathways in Fig 8. Human and mouse relative biomarker concentrations, fold differences (log2) of median value of CM relative to NCM, in each pathway were compared. Activated biomarkers in NCM phenotype of both species are marked in blue, and activated biomarkers in CM phenotype of both species are marked in orange. Download Table S1, XLSX file, 0.8 MB. Copyright © 2022 Cha et al. 2022 Cha et al. https://creativecommons.org/licenses/by/4.0/ This content is distributed under the terms of the Creative Commons Attribution 4.0 International license.

We also analyzed blood stage plasma biomarker activation pattern at 6 dpi, which is 2–3 days before CM development (see Fig. 1A) (Fig. 3; Table S1A, column AW in the supplemental material). Angiogenesis, chemotaxis, and anti-apoptosis markers were activated in NCM mice (Fig. 3, right middle panel, Fig. S3B) and five pro-inflammatory, leukocyte infiltration, pro-apoptosis markers were activated in CM mice (Fig. 3, right lower panel, Fig. S3C). Less stringent statistics identified additional blood stage biomarkers showing significant differences between CM and NCM phenotypes (Fig. S4B, C; Table S1A, columns BG). These patterns strongly imply that Th1 immunity is activated at blood stage in CM mice while this is not the case for NCM mice, possibly because of inhibition by Th2 immune factors that were activated during liver stages. Importantly, NCM mice of both genotypes (WT and CD68 KO) uniquely activate angiogenesis markers (DLL4 and Cystatin C), as well as an anti-apoptotic marker (IGF-1) that stabilize the blood brain barrier (Fig. 3; Fig. S3B, Fig. S4B) (20–22).

### Sporozoite infection of the CD68 KO liver induces injury.

**FIG 4:**
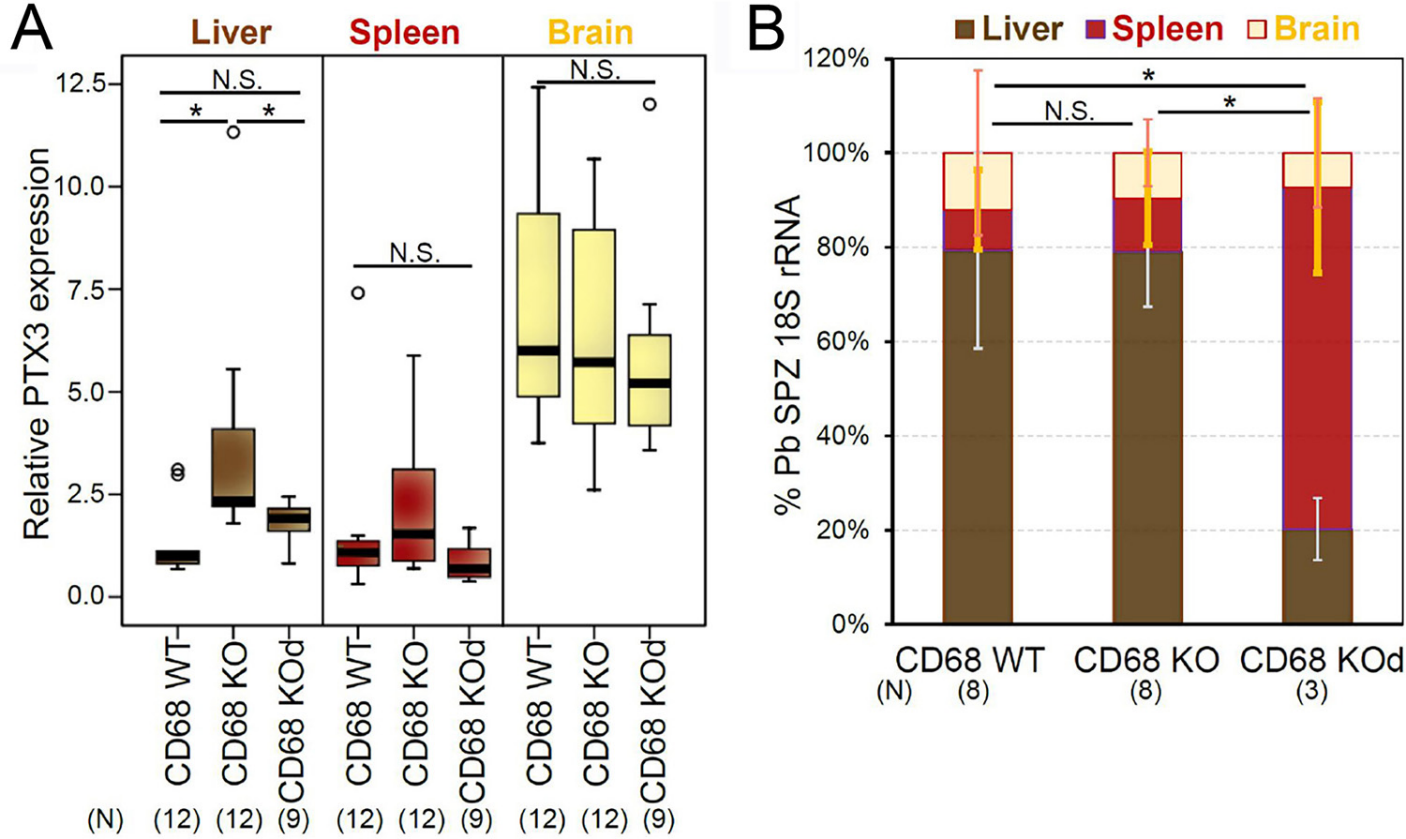
Hepatic vascular injury model as it relates to the alternative sporozoite modes of exit from the hepatic circulation. To identify the organ responsible for vascular injury, 3 × 10^4^ live or dead sporozoites were injected into the tail vein of WT and CD68 KO mice. Total RNA was isolated from liver, spleen, and brain at 4 h after injection. (A) Quantification of expression of the PTX3 vascular injury marker gene in the three organs. All box plots show quartiles, median, and maximum. (B) Distribution of parasite rRNA among the three organs. Error bars show standard deviations. (A, B) Data pooled from two independent experiments. CD68 KOd: CD68 KO mice injected with dead sporozoites. *N*, total number of mice assayed. *P* value was calculated with the Mann-Whitney U test. *P* values (* ≤0.05). N.S., not significant.

The unique liver stage NCM plasma biomarker activation pattern (Fig. 3; Fig. S3A, 4A) suggested that it is connected to hepatic vascular injury during sporozoite invasion of the CD68 KO liver. To test this hypothesis, we measured expression of the PTX3 injury marker in the liver, spleen, and brain of WT and CD68 KO mice at 4 h after sporozoite infection. PTX3 expression is known to be quickly activated upon injury and to peak at 6 h (23). We found that PTX3 expression is significantly activated specifically in CD68 KO liver, not in the WT liver, nor in the spleen or in the brain of CD68 KO mice (Fig. 4A). This activation was not observed at 2 dpi (Table S1A, columns AT, BD in the supplemental material) as expected, since this is beyond the transient PTX3 activation profile (23). At 4 h postinfection, around 80% of the live sporozoites were found in the liver while ∼80% of dead sporozoites were cleared by the spleen, also as expected (Fig. 4B). These results show that sporozoite liver infection triggers the activation of the acute injury marker PTX3 only in CD68 KO mice, not in WT mice. CD68 KO mice lack the sporozoite receptor for vascular traversal through Kupffer cells.

### IGF-1 inhibits CM development.

**FIG 5:**
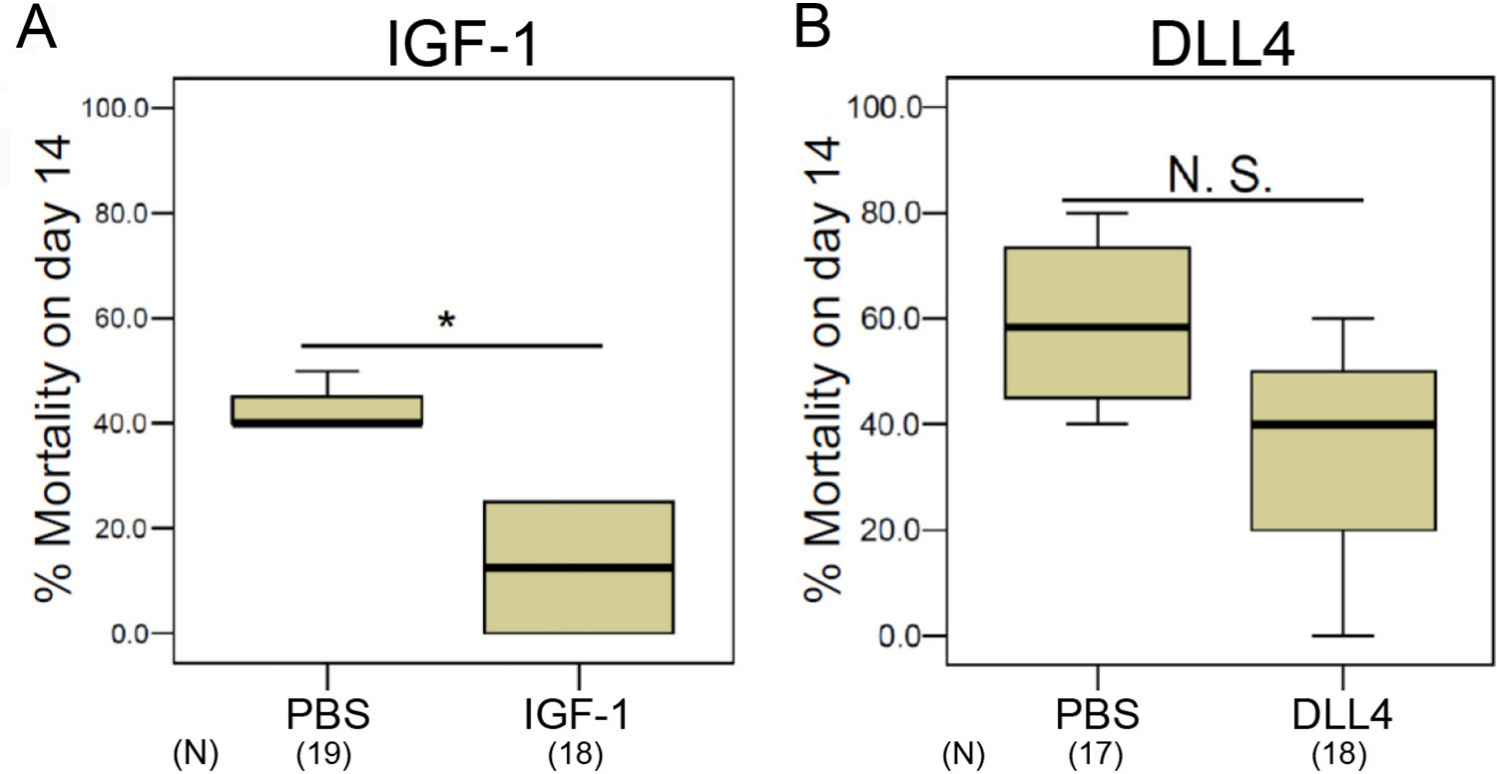
IGF-1 inhibits CM development in sporozoite infected WT mice. We injected recombinant (A) IGF-1 (2 μg per mouse) or (B) DLL4 (120 ng per mouse, right panel) in PBS into WT mice after infection with 2,000 sporozoites. The injected doses were determined as five times physiological concentration in plasma. Daily intravenous injections were performed from days 3 to 10 postinfection. PBS alone served as a control. Percent mortality on day 14 with IGF-1 (left panel) or DLL4 (right panel) treatment shows IGF-1 significantly inhibits CM development. Data pooled from four independent experiments. (N): total number of mice assayed. All box plots show quartiles, median, and maximum. *P* value was calculated with the Mann-Whitney U test. *P* values (* ≤0.05). N.S., not significant.

Among the blood-stage inhibition markers were IGF-1 and DLL4 (Fig. 3, right middle panel). IGF-1 is a Th2 immunity component and a well-known survival factor that stabilizes BBB integrity (22). IGF-1 inhibits tumor necrosis factor-α (TNF-α), which is a key inflammatory CM activator (5). DLL4 is the dominant Notch signaling ligand (angiogenesis inducing marker) that triggers activation of the IL-33 pathway, a key player in CM inhibition (17, 24, 25). To investigate whether IGF-1 and/or DLL4 can by themselves inhibit CM development, sporozoite-infected WT mice were injected daily with recombinant mouse IGF-1 or recombinant mouse DLL4 during days 3 to 10 postinfection. As shown in Fig. 5A, IGF-1 injection significantly reduced CM-associated mortality in WT mice while injection of DLL4 also reduced mortality, but this decrease was not statistically significant (Fig. 5B).

### Connection between hepatic vascular injury and CM development.

**FIG 6:**
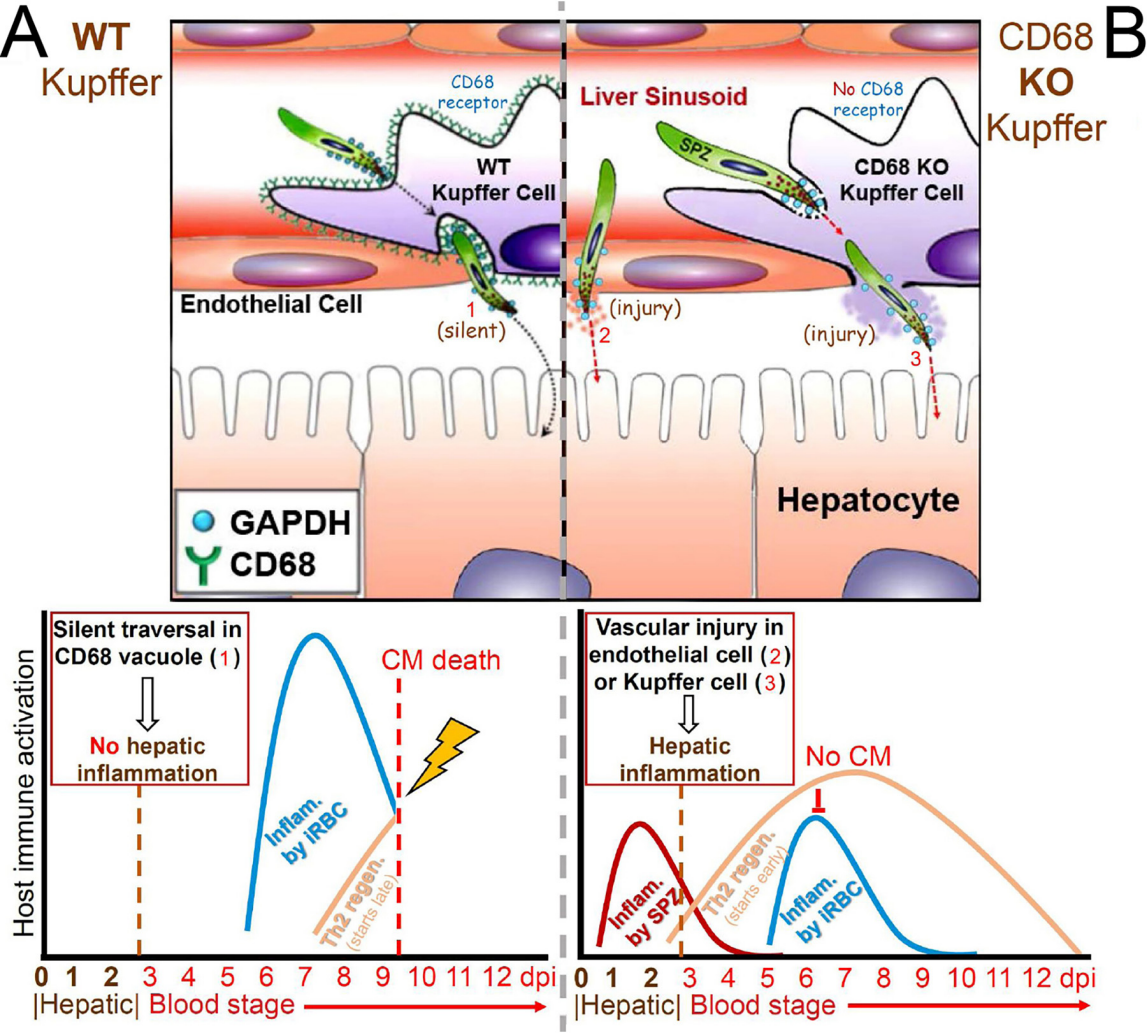
Hepatic vascular injury model of CM development and inhibition influenced by alternative pathways for sporozoite exit from the hepatic circulation. Sporozoites in the WT mouse exit the circulation “silently” without triggering host cell immunity, by traversing Kupffer cells in a CD68-coated vacuole (A upper panel). On the other hand, in the CD68 KO mouse sporozoites traverse Kupffer cells lacking the CD68 receptor or endothelial cells, by breaching these cells and triggering hepatic vascular injury (B upper panel). CM development pathway in the WT mouse (A, lower panel): CD68-dependent sporozoite exit from the circulation occurs silently, without vascular injury. Later, iRBC attachment to brain vessels cause sequestration of activated immune cells while iRBC clearance in the spleen triggers inflammation (blue curve). Proinflammatory Th1 immunity causes cerebral vascular injury and consequent CM-associated mortality, before modulation by the follow-up Th2 regeneration process can take place (A lower panel); CM inhibition pathway in the CD68 KO mouse (B, lower panel): Injury caused by CD68-independent sporozoite liver invasion triggers early Th1 immune activation (red curve), inducing early downstream activation of the Th2 regeneration process (yellow line) that inhibits later inflammation and associated CM development.

Our results are compatible with a hepatic vascular injury model that shows how two alternative sporozoite-liver invasion modes modulate CM development (Fig. 6). When the CD68 sporozoite receptor is present on the surface of Kupffer cells, sporozoites leave the circulation “silently” via transcytosis, a process that minimizes immune activation (Fig. 6A upper panel) (7). Conversely, in the absence of the CD68 receptor, sporozoites reach the liver by cell ‘traversal’ that breaches the blood vessel cell lining and causes vascular injury (Fig. 6B upper panel) (24). The silent CD68-dependent sporozoite liver infection is later followed by blood stage Th1 immune activation (KC, INFγ, MIG, TCA-3, RANTES, and MCP-5) and subsequent pro-apoptotic activation (TWEAK and Granzyme B) which results in BBB disruption-related fatality before the Th2 regeneration phase can take place (Fig. 6A lower panel; see also Fig. 3 and Fig. S3C, 4C) (9, 24, 26–28). In the absence of the CD68 receptor, hepatic vascular injury triggers early Th1 immunity, followed by Th2 immunity activation that dampens Th1 immunity and protects mice from fatal CM (Fig. 6B; lower panel) (29). Furthermore, Th2 immunity, including anti-apoptotic (IGF-1) and angiogenesis factors (DLL4 and Cystatin C) in NCM mice, maintains BBB integrity (Fig. S1B).

### Comparative study of human and mouse CM.

**FIG 7:**
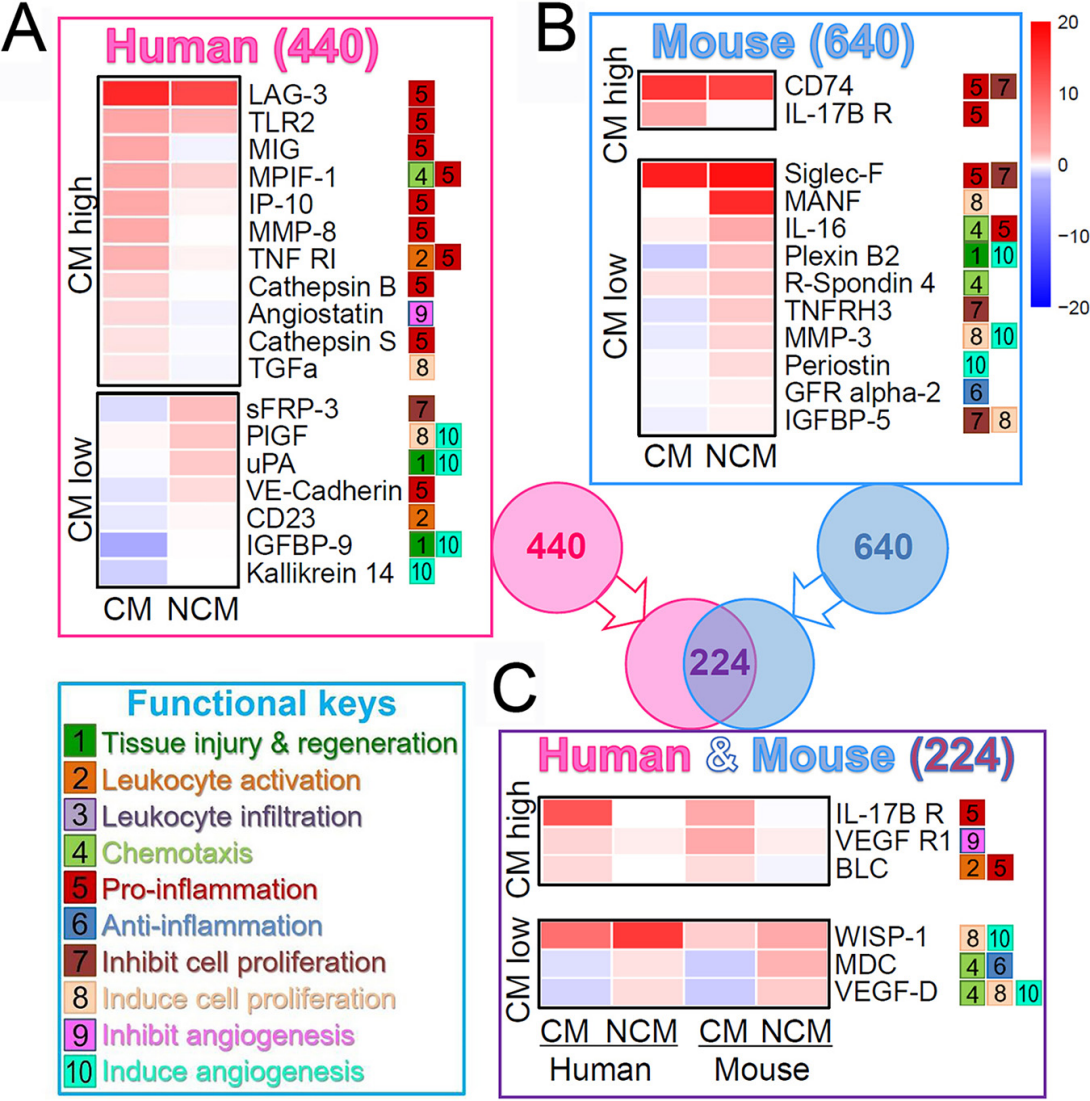
Comparative human and mouse plasma biomarker analysis for CM development and inhibition pathways. (A) Heat map of multiplex ELISAs of plasma collected from CM or NCM (mostly asymptomatic) pediatric malaria-infected subjects. Eighteen out of 440 biomarkers tested showed significant difference (TukeyHSD test, *P < *0.05) between CM and NCM patients. (B) Heat map of multiplex ELISAs of plasma collected from CM or NCM WT mice 10 days (equivalent time point to human CM development) after infection with 2,000 sporozoites. Twelve out of 640 biomarkers tested showed significant difference (TukeyHSD test, *P < *0.05) between CM and NCM phenotypes. (C) Heat map of multiplex ELISAs of human and mouse plasma showing similar trends. Six out of 224 biomarkers tested show more than 1.5-fold difference between two CM-related phenotypes. Two-way ANOVA test excluded biomarkers showing significant difference between human and mouse. (*A–C*) Color-coded fold differences (log_2_) of median relative to uninfected human or mock treated mouse controls. Identified biomarkers were further classified into two groups: CM high (higher concentration in CM than in NCM) or CM low (lower concentration in CM than in NCM), and functional keys denote known functions of each biomarker. Data pooled from two or four independent repeats.

Retroactive deduction of CM-associated biomarker activation is not feasible to investigate human CM because plasma samples from the same individual collected before and after phenotype development are not available. To examine to what degree mouse data can be extended to humans, we performed comparative plasma assays with samples collected from mouse and humans after phenotype development. Mouse CM and mortality occurs mainly on 9–10 dpi. We collected and analyzed plasma from CM and NCM WT mice on day 10 after injection of 2,000 sporozoites. For humans, we used plasma collected from Ugandan children with CM or with asymptomatic P. falciparum parasitemia (NCM) (Table S1B in the supplemental material) (30). One-way ANOVA test identified 18 human CM-associated biomarkers, out of 440 markers tested, that show significantly different activation patterns between CM and NCM patients (Fig. 7A; Fig. S5, Table S1C in the supplemental material), and 12 mouse biomarkers, out of 640 markers tested (Fig. 7B; Fig. S6, Table S1D). These biomarkers were further sorted into upregulated (CM-high) or downregulated (CM-low), relative to NCM subjects (Fig. 7A and B). Pro-inflammatory and pro-apoptotic biomarkers dominate the CM-high human and mouse groups, whereas anti-inflammatory, anti-apoptotic, and angiogenesis markers dominate the CM-low groups.

10.1128/mbio.03708-21.5 FIG S5 Identification of plasma biomarker groups associated with human CM-related phenotypes. Eleven plasma biomarkers are up-regulated in human CM subjects, and four markers are up-regulated in NCM human subjects. Three markers are down regulated in human CM subjects. UI: uninfected. *P* value was calculated with one-way ANOVA TukeyHSD test. *P* values (* ≤0.05, ** ≤0.01, *** ≤0.001). Download FIG S5, JPG file, 1 MB. Copyright © 2022 Cha et al. 2022 Cha et al. https://creativecommons.org/licenses/by/4.0/ This content is distributed under the terms of the Creative Commons Attribution 4.0 International license.

10.1128/mbio.03708-21.6 FIG S6 Identification of plasma biomarker groups associated with WT mouse CM-related phenotypes. Two plasma biomarkers are up-regulated in CM mice, and nine markers are up-regulated in NCM mice. One marker is down regulated in CM mice. UI: uninfected. *P* value was calculated with one-way ANOVA TukeyHSD test. *P* values (* ≤0.05, ** ≤0.01, **** ≤0.0001). Download FIG S6, JPG file, 0.9 MB. Copyright © 2022 Cha et al. 2022 Cha et al. https://creativecommons.org/licenses/by/4.0/ This content is distributed under the terms of the Creative Commons Attribution 4.0 International license.

A separate analysis found that 224 biomarkers are common between mouse and human plasma assays. Of these, two-way ANOVA test identified six that show conserved activation patterns in both species with greater than 1.5-fold difference (Fig. 7C; Fig. S7, Table S1E in the supplemental material). Also here, CM-high markers are involved in pro-inflammatory immunity and CM-low markers are involved in anti-inflammatory and angiogenesis processes. In particular, the data suggest that IGF-1 activation is involved in the CM-inhibitory pathway both in humans and mice. This is because downstream markers—human IGFBP-9 (IGF-1-Binding Protein-9) and mouse IGFBP-5 and MMP3—are enriched in NCM plasma of both humans and mice (Fig. 7A and B; Fig. S5–S7, Table S1C to E) (18, 30–33).

10.1128/mbio.03708-21.7 FIG S7 Identification of conserved plasma biomarker groups in human and mouse associated with CM-related phenotypes. Three plasma biomarkers are up-regulated in CM-positive mice and human subjects, and one marker is up-regulated in NCM mice and human subjects. Two markers are down-regulated in NCM mice and human subjects. Data pooled from two or four independent repeats. UI: uninfected. *P* value was calculated with one-way ANOVA TukeyHSD test. *P* values (* ≤0.05). Download FIG S7, JPG file, 1 MB. Copyright © 2022 Cha et al. 2022 Cha et al. https://creativecommons.org/licenses/by/4.0/ This content is distributed under the terms of the Creative Commons Attribution 4.0 International license.

**FIG 8:**
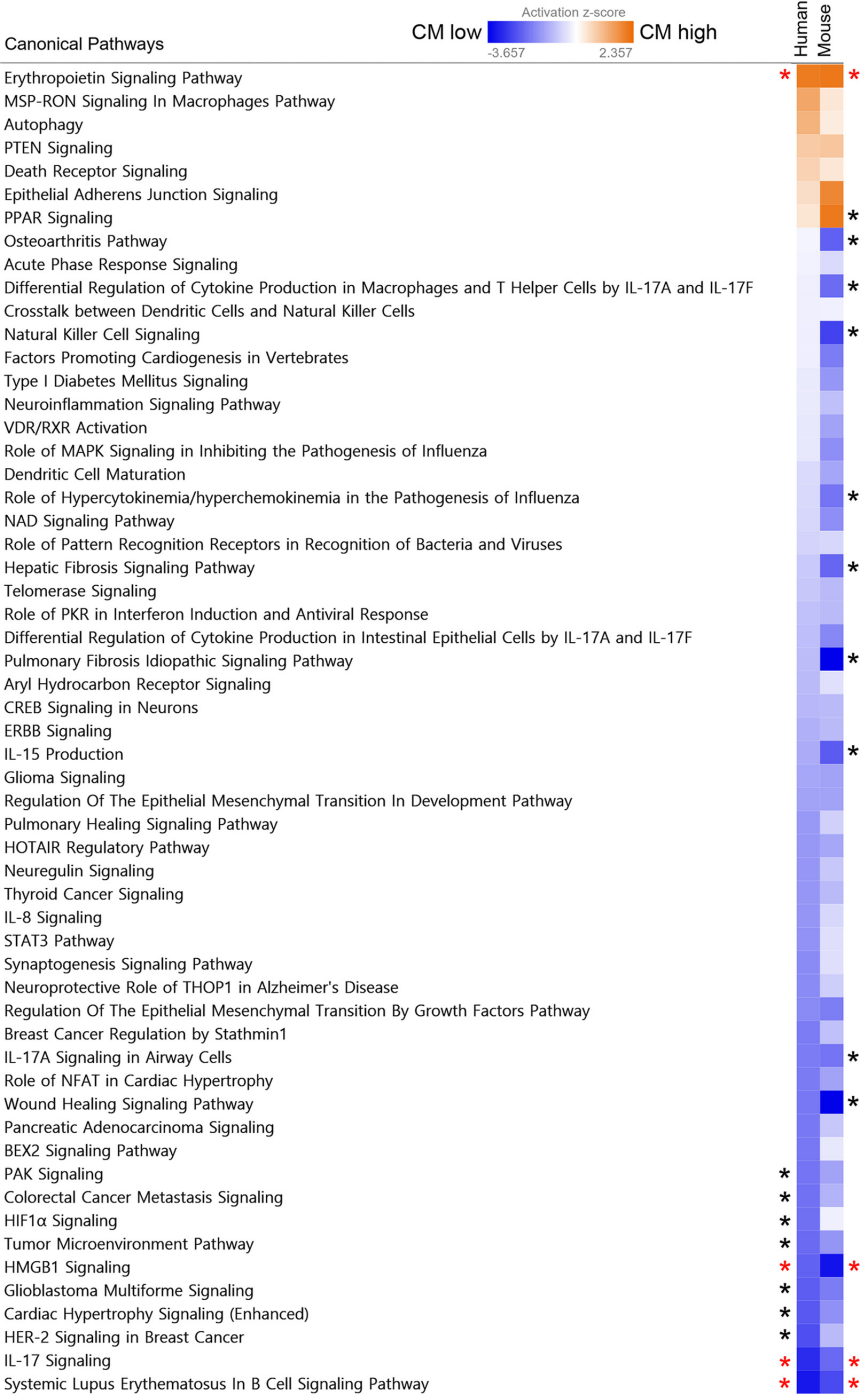
Canonical CM-related pathway comparison between human and mouse plasma factors. Human and mouse plasma biomarker median concentrations in Tables S3 and S4 in the supplemental material were analyzed for canonical pathway activation profiles. The heat map shows conserved human and mouse canonical pathway activation profiles. Significantly activated pathways in CM phenotype (CM high, activation z score 2 and over) and significantly activated pathways in NCM phenotype (CM low, activation z score -2 and below) in either species are marked with black asterisks and significantly activated pathways in both species are marked with red asterisks.

To supplement the comparison of the 224 biomarkers mentioned above, we expanded the canonical pathways analysis to the 440 human and 640 mouse markers tested. We identified 58 pathways related to CM development that are conserved between humans and mice (Fig. 8). The erythropoietin signaling pathway is significantly upregulated in CM-positive human and mouse plasma, whereas HMGB1, IL-17, and systemic lupus erythematosus in B-cell signaling pathways were significantly downregulated in CM-positive plasma of both species. Human and mouse biomarker activation profiles belong to these four pathways are shown in Table S1F to I in the supplemental material.

## DISCUSSION

Our study shows that CM development is not determined by parasite numbers but rather, by how sporozoites exit the liver circulation, which in turn modulates host immune pathways. Previously, intravital video microscopic studies with intravenous injection of 3 × 10^5^ sporozoites showed that to exit the circulation in the liver, the majority of sporozoites use an alternate cell ‘traversal’ machinery by breaching the blood vessel cell lining (34). However, qRT-PCR assays with 2 × 10^3^ sporozoite injection show that liver invasion of CD68 KO mice is reduced by ∼70% compared with WT controls (2), which implies that lower-dose, natural-like infection mainly relies on the CD68-dependent transcytosis. We show that the CM-resistant phenotype of CD68 KO mice is mediated by soluble plasma factors generated during liver stage infection, independent of sporozoite numbers used for the challenge. CM development is not determined by blood stage parasite burden. Presumably, parasite multiplication in the blood circulation is controlled by host immunity after merozoites are released from the liver. Some mice had high parasitemia and did not develop CM but died of severe anemia at around 30 dpi. This is analogous to human malaria patients who can become severely anemic without developing CM (3).

Our plasma assays show that CD68-independent sporozoite traversal induces hepatic vascular injury, which further triggers anti-inflammatory type 2 immune activation for vascular regeneration. Although sporozoite-infected hepatocytes activate INFγ-mediated inflammation (3), no type 2 immunity is activated in CM-positive mice at 2 dpi. Our model is supported by the observation that stool helminth infections decrease the risk of CM in children with malaria (35, 36); helminths activate Th2 immunity that leads to Th1 immunity inhibition. Our findings are also in agreement with recent data showing that liver invasion by transgenic P. berghei sporozoites possessing increased cell transversal activity (and presumably increased cell injury), strongly inhibits CM development (37).

The day-6 activation of DLL4 and IGF-1 in CM-negative mice provides examples of predictive biomarker of CM inhibitory pathway. Beside angiogenesis activation, DLL4 is the dominant Notch signaling ligand that triggers activation of the IL-33 pathway, a key player in mouse CM inhibition (4, 5). IGF-1 is mainly generated in the liver and circulating IGF-1 is absorbed by the brain endothelial cells (6). IGF-1 has been recognized as a potent and wide-spectrum neuroprotective agent in all types of brain injuries (38). In the brain, IGF-1 inhibits neurodegeneration with pro-survival effects on damaged neurons through the PI3K-AKT pathway, which inhibits oxidative stress and apoptosis. Moreover, IGF-1 protects neurons against maladaptive inflammation by inhibiting TNF-α, a common underlying process in neurodegeneration, which is also known to be important for CM development (6–8).

Whereas during liver stages (day-2 postinfection) IGF-1 plasma levels of uninfected, CM and NCM mice are the same, on day-6 there is a significant drop of IGF-1 levels only in mice that develop CM. We hypothesize that reversal of this IGF-1 drop in mice that do not develop CM is triggered by vascular injury during sporozoite receptor-less invasion of the liver. In accordance with this hypothesis, early administration (starting on day-3) of IGF-1 precludes the drop and in this way protects mice from CM. At late blood stages (day-10) IGF-1 plasma levels of both CM and NCM mice are low compared to uninfected controls, predicting that late IGF-1 administration would be inconsequential for CM occurrence. By which mechanism the presence of IGF-1 during early blood stages prevents CM occurrence is a matter that requires further investigation.

Our comparative studies suggest human CM inhibitory pathway also relies on IGF-1-mediated type 2 immune activation because both human and mouse NCM plasmas have higher IGFBP level, which is induced by IGF-1. The previous finding that human IGFBP-1 is a severe malaria anemia (SMA) marker is in agreement with our results because SMA develops in CM negative patients (6, 39). Although human plasma samples are not completely equivalent to the mouse day-10 plasma samples, decreased IGFBP level in human and mouse CM plasma suggests that inhibition of the IGF-1 pathway can serve as a CM predictive human biomarker. In addition to IGF-1, our mouse day 6 plasma analysis identified further CM predictive biomarkers: two decreased biomarkers (DLL4, C5a) and five increased biomarkers (INFγ, MIG, MCP-5, Granzyme B, and TWEAK). We expect that these biomarkers will help predict human CM.

In summary, we report on two aspects of CM development after *Plasmodium* infection. First, the mode of sporozoite exit from the liver circulation (by silent transcytosis or by breaching cells that line the blood vessels) is consequential for CM development. Why do some WT mice develop CM and others not? Cell breaching occurs also in the wild type mouse (34), and most likely the balance between silent traversal and breaching influences CM fate. Second, CM is downregulated by early activation of protective Th2 immunity triggered by sporozoite hepatic vascular injury. This report for the first time connects alternate *Plasmodium* liver invasion pathways to the expression of prognostic biomarkers of CM. These findings are expected to lead to the development of novel preventive and therapeutic treatments of human CM.

## MATERIAL AND METHODS

### CD68 KO mouse and *Plasmodium* infection.

The CD68 KO mouse was originally generated using 129 R1 ES cells, and then backcrossed seven times to C57BL/6 mice (40). This mouse was transferred to Johns Hopkins Bloomberg School of Public Health and maintained under a protocol approved by the Johns Hopkins University Animal Care and Use Committee. F_2_ knockout homozygotes were used as the CD68^−/−^ KO line and used CD68^+/+^ from the same lineage as a WT control line. Our P. berghei-infected control mice frequently display a number of CM-related symptoms such as ruffled fur, hunching, wobbly gait, limb paralysis, convulsions and coma (4), suggesting that the seven backcrosses restored the typical CM phenotype of C57BL/6 mice. CM-associated phenotypes develop at 8∼13 dpi; once an infected mouse develops CM-associated symptoms, it dies within 24 h (Fig. 1A). We defined these lethal cases that occur within 14 dpi as CM. Mice that survive 14 dpi generally die around 30 dpi of severe anemia. BBB disruption of CM-positive mice was visualized by Evan’s Blue infusion assay: 100 μl of 2% Evan’s Blue in PBS was injected into their tail vein. After 50 ml of PBS perfusion throughout the body, the brain was isolated from CM-negative (NCM) mice or CM-positive (CM) mice and photographed. Incidence of the CM development in WT or CD68 KO mice does not differ between sexes (Fig. S8 in the supplemental material).

10.1128/mbio.03708-21.8 FIG S8 CM incidence in WT and CD68 KO mice is sex-independent. CM incidence was significantly higher for WT mice than for CD68 KO mice of both sexes. Female mice of each genotype show higher CM incidence than male mice, however this difference is not statistically significant. Data pooled from six independent experiments. *P* value was calculated with the Mann-Whitney U test. *N*, total number of mice assayed. All box plots show quartiles, median, and maximum. *P* values (* ≤0.05; ** ≤0.01). N.S., not significant. Download FIG S8, JPG file, 0.5 MB. Copyright © 2022 Cha et al. 2022 Cha et al. https://creativecommons.org/licenses/by/4.0/ This content is distributed under the terms of the Creative Commons Attribution 4.0 International license.

P. berghei ANKA sporozoites were isolated from infected Anopheles stephensi mosquitoes using salivary gland dissection. Mock-preps were prepared with the same isolation procedures, but from uninfected mosquitoes. Mice were infected by intravenous injection of sporozoites or iRBCs, or by mosquito biting. Dead sporozoites (Fig. 3) were prepared by repeated freezing and thawing.

### Plasma transfer assay.

WT or CD68 KO mice received intravenous injection of 20,000 P. berghei sporozoites (WT or SPECT2 KO) or mock-preparations isolated from an equivalent number of uninfected mosquitos. After 45 h (2 dpi), before the onset of blood infection, we collected plasma and injected 100 μl into WT mice. One day after plasma transfer, mice were infected by intravenous injection of 20,000 iRBCs and CM development was followed up to day 14. Injection of plasma from PBS-injected CD68 KO mice did not alter CM development of recipient mice (data not shown).

### Multiplex ELISA.

Either 2,000 P. berghei sporozoites or mock-preparations from uninfected mosquito salivary glands were injected through the tail vein of WT or CD68 KO mice. At 2 and 6 dpi, ∼50 μl plasma was collected from each mouse (plasma from mock-prep treated mice was collected only at 2 dpi). Plasma samples were preserved at −80°C until CM-associated phenotyping at 14 dpi and were further assorted into groups according to the time of collection (2 or 6 dpi), according to CD68 genotype, and according to CM phenotype. A total of 5∼6 plasma samples of each group were pooled for Quantibody Array ELISA analysis (Ray Biotech, Inc.). Each biomarker concentration (pg/ml) was determined using multiplex ELISAs accompanied with standard curve reactions. Statistical analysis was performed with data pooled from four independent repeats of mock-treated CD68 KO plasma, CM-positive WT plasma at 2 dpi, CM-negative CD68 KO plasma at 2 dpi and with data from two independent repeats for the other cases.

### Human plasma samples.

We assayed human plasma samples collected from CM-positive and CM-negative pediatric Ugandan malaria patients as previously described (Table S1B in the supplemental material). Human CM was defined as: i) coma (Blantyre Coma Score <2); ii) Plasmodium falciparum on blood smear; and iii) no other known cause of coma (e.g., meningitis, a prolonged postictal state or hypoglycemia-associated coma reversed by glucose infusion). Written informed consent was obtained from parents or guardians of study participants. Ethical approval was granted by the Institutional Review Boards for human studies at Makerere University School of Medicine and the University of Minnesota. Details of human plasma sample collection were described previously (30). We used 40 human plasma samples (50 μl/sample) as follows: 20 CM-positive (CM) samples, 10 CM-negative samples (NCM, all with asymptomatic P. falciparum parasitemia, none of whom had prior CM or developed CM over 2 years of follow-up), and 10 uninfected control (UI) plasma samples, collected from 21 male and 19 female children 1.8∼11.4 years-old (Table S2). To attain minimum volume for Quantibody Array ELISA analysis (Ray Biotech, Inc.), five samples per group were pooled, for a 250 μl/pool, yielding 4 CM samples and two NCM and two UI samples.

### CM inhibition assay using recombinant mouse DLL4 and IGF-1.

WT mice were infected by IV injection of 2,000 sporozoites. From day 3 to 10, infected mice received daily injections of 50 ng of recombinant mouse DLL4 (R&D Systems, 1389-D4) or 2 μg of recombinant mouse IGF-1 (R&D Systems, 791-MG). Injection dose of each plasma biomarker was determined as five times of physiological concentration in Fig. S3B in the supplemental material. PBS injection served as a control. CM-associated mortality was determined on day 14 after sporozoite infection.

### Pathway analysis.

Mouse and human plasma biomarker activation profiles determined by multiplex ELISAs (Table S1C and D in the supplemental material) were further compared using Ingenuity Pathway Analysis (https://digitalinsights.qiagen.com/products-overview/discovery-insights-portfolio/analysis-and-visualization/qiagen-ipa/) to identify conserved CM-related pathways between human and mouse. Fold differences (log_2_) of median value of CM relative to NCM were used for canonical pathway analysis using Core Analysis tool.

### Statistical analyses.

To perform multiple comparison analyses, we used the one-way ANOVA in the R Stats package followed by computing Tukey Honest Significant Differences using the R function TukeyHSD. Firstly, for each of the 10 groups, the genotype-specific biomarkers that were significantly different (adjusted *P* value <0.05) between WT and KO in mock, day 2 CM, day 2 no CM, day 6 CM, or day 6 no CM were filtered. Subsequently, samples for different genotypes that displayed the same phenotypes were merged into a total of five groups for identifying phenotype-specific biomarkers (adjust *P* value <0.05) using the ANOVA-based approach followed by TukeyHSD as described directly above (Table S1A and C to E and Fig. S3 in the supplemental material). Finally, the fold change of biomarkers in each sample compared to mock were visualized as a heat map with 5 customized clusters by using the R function pheatmap (Fig. 3 and 7).

For the multiplex ELISA, we pooled 50 μl plasma samples from five mice in a group, which reduced sample number for statistical analysis. Therefore, we expected to have a fair amount of false negatives, and we additionally filtered out moderately significant biomarker activations (*P* value <0.05) using one-way ANOVA-based analyses by computing Fisher's Least Significant Difference (LSD) *post hoc* test from the non-significant candidates using the TukeyHSD test (Table S1A; Fig. S4 in the supplemental material). We used the two-way ANOVA test for comparison between mouse and human, between CM and NCM plasma samples (Fig. 7C; Fig. S7).
