# Supplementary figures and images for: Identification of Key Determinants of Cerebral Malaria Development and Inhibition Pathways
Source: mBio. 2022 Jan 25;13(1):e03708-21. doi: 10.1128/mbio.03708-21 (PMC8787489; doi:10.1128/mbio.03708-21)

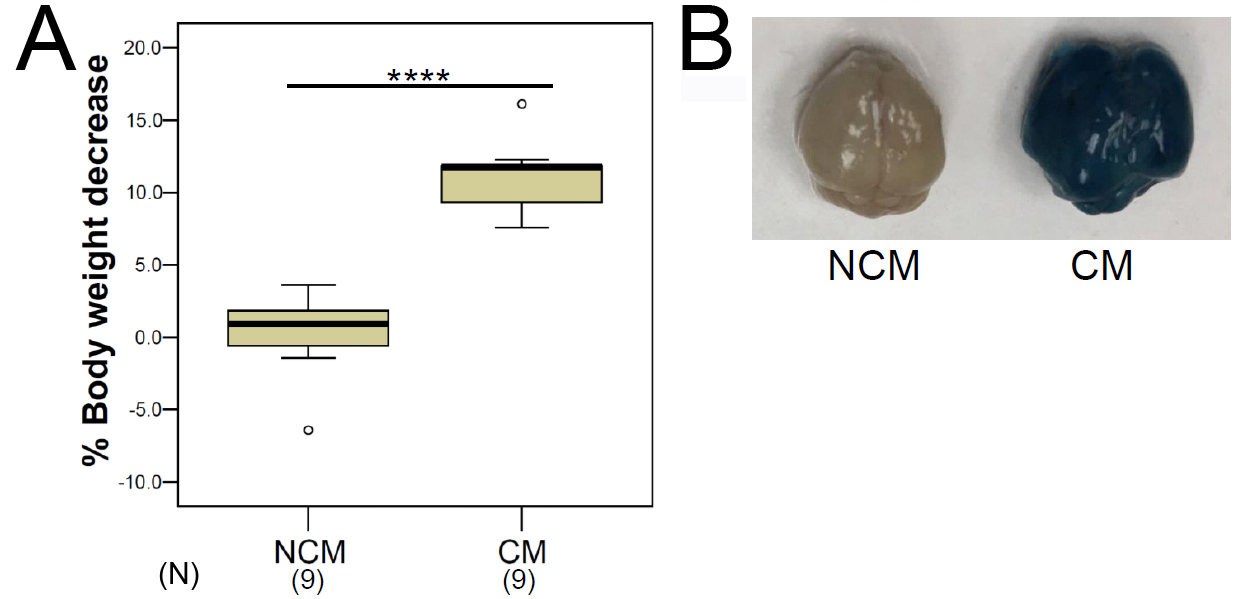

Supplement: FIG S1 [file mbio.03708-21-sf001.tif]

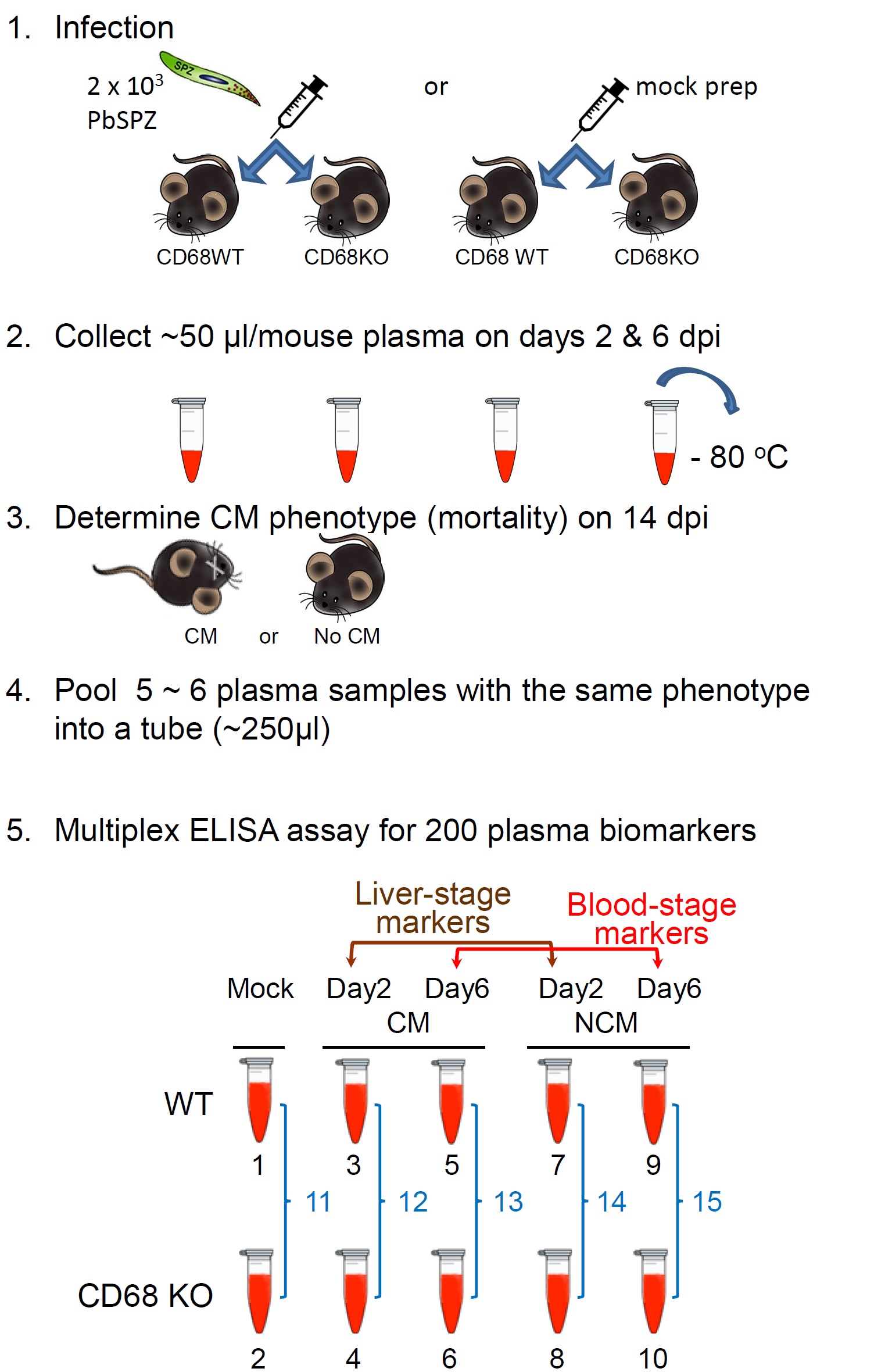

Supplement: FIG S2 [file mbio.03708-21-sf002.jpg]

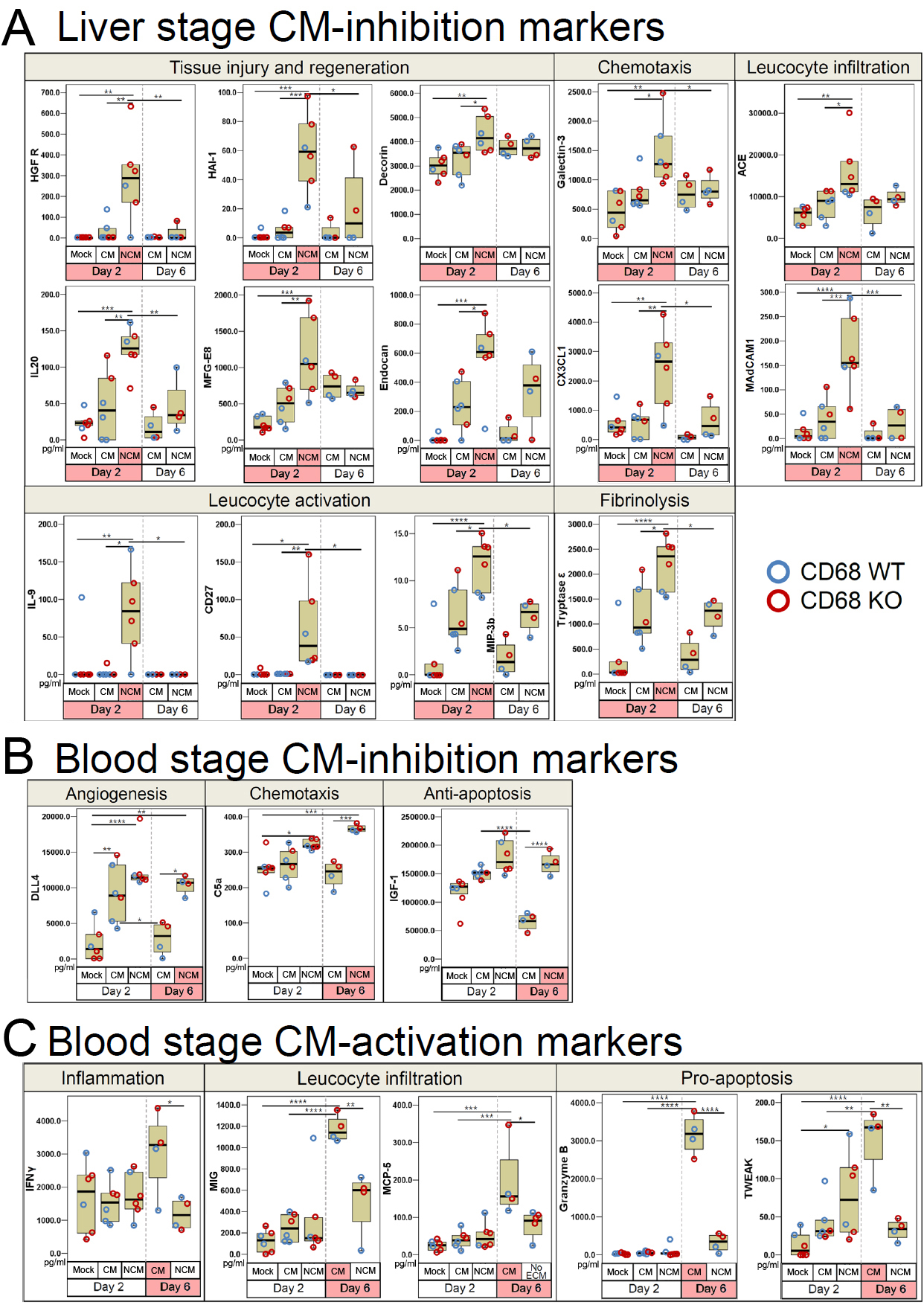

Supplement: FIG S3 [file mbio.03708-21-sf003.jpg]

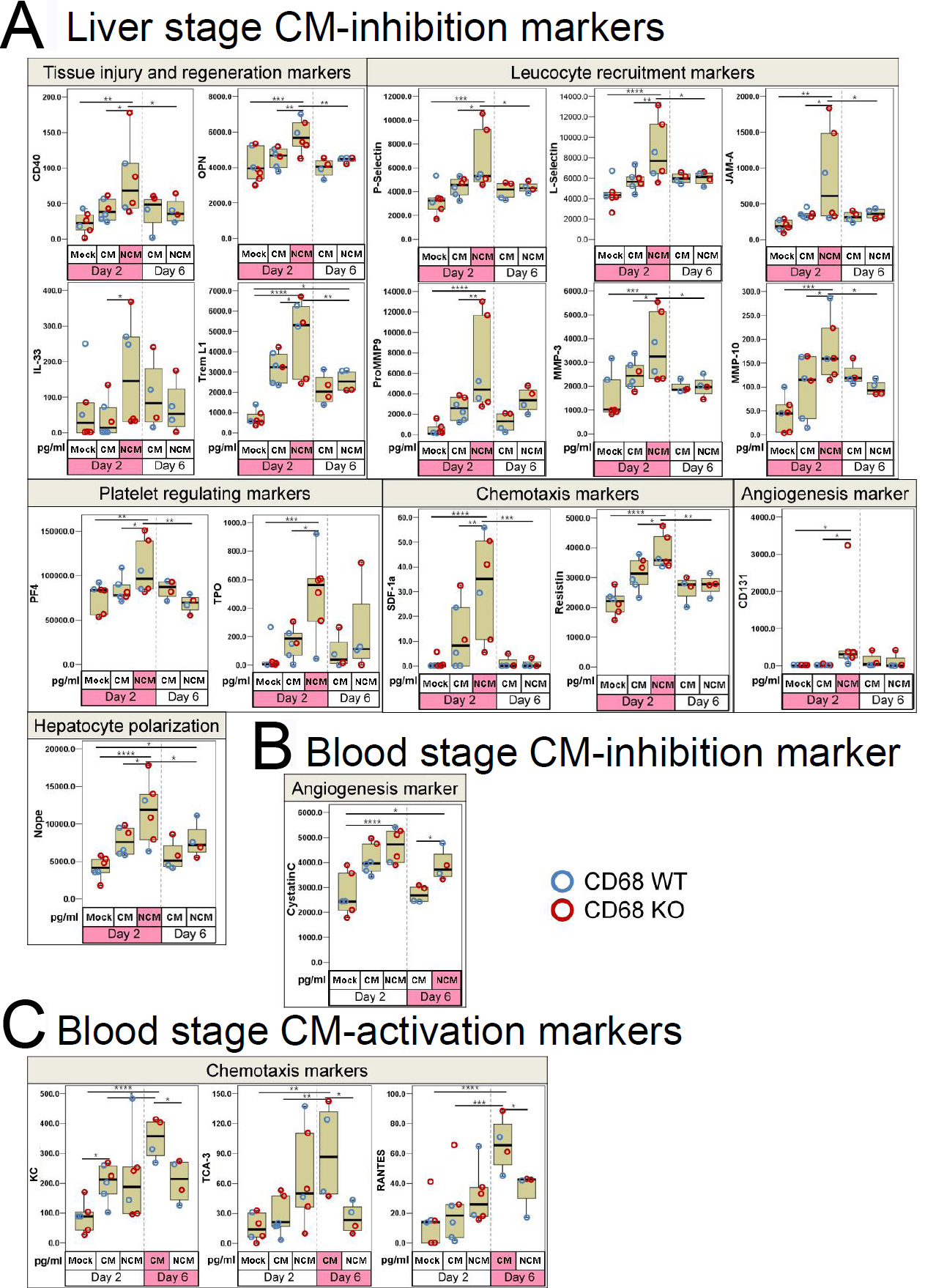

Supplement: FIG S4 [file mbio.03708-21-sf004.jpg]

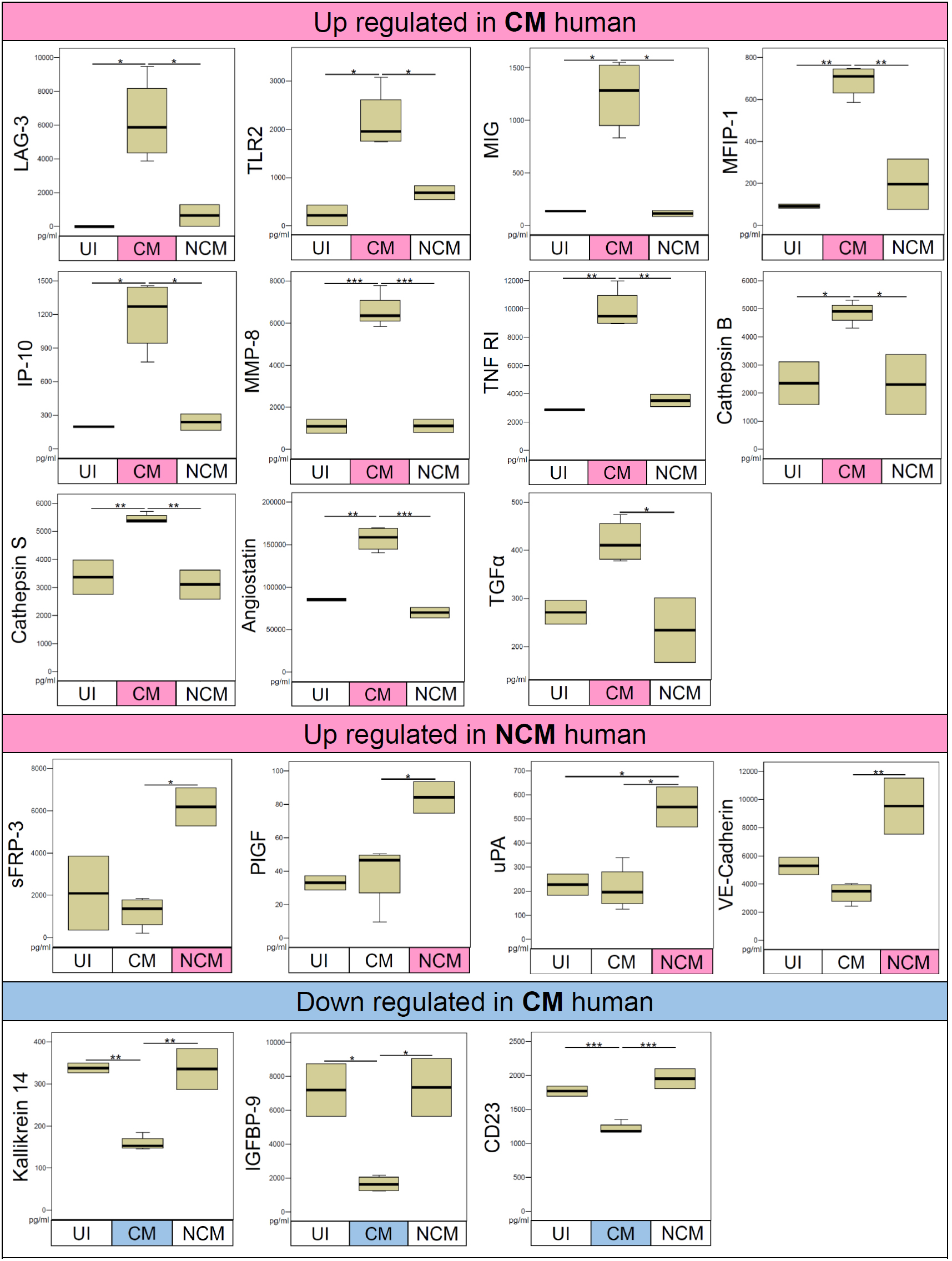

Supplement: FIG S5 [file mbio.03708-21-sf005.jpg]

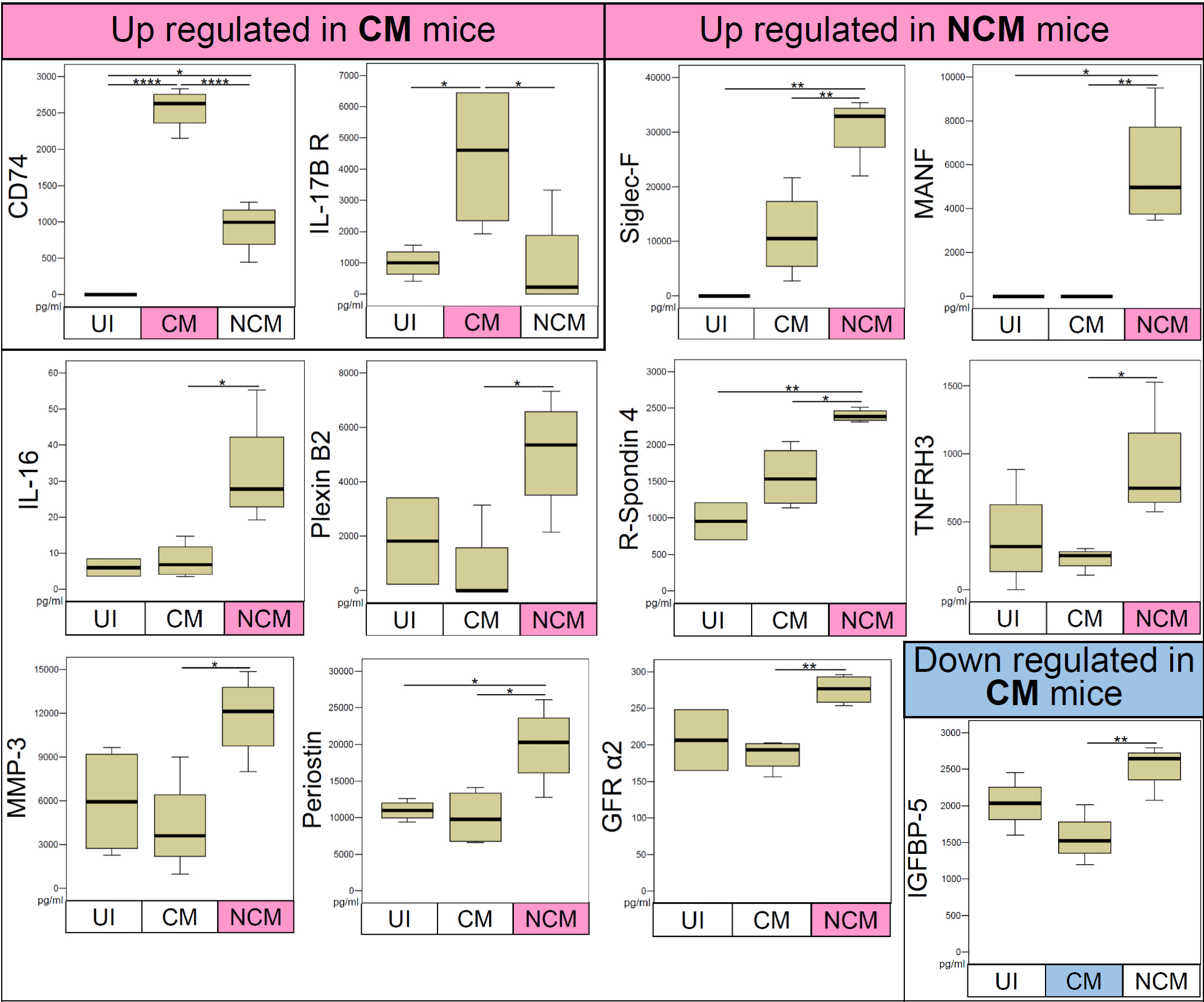

Supplement: FIG S6 [file mbio.03708-21-sf006.jpg]

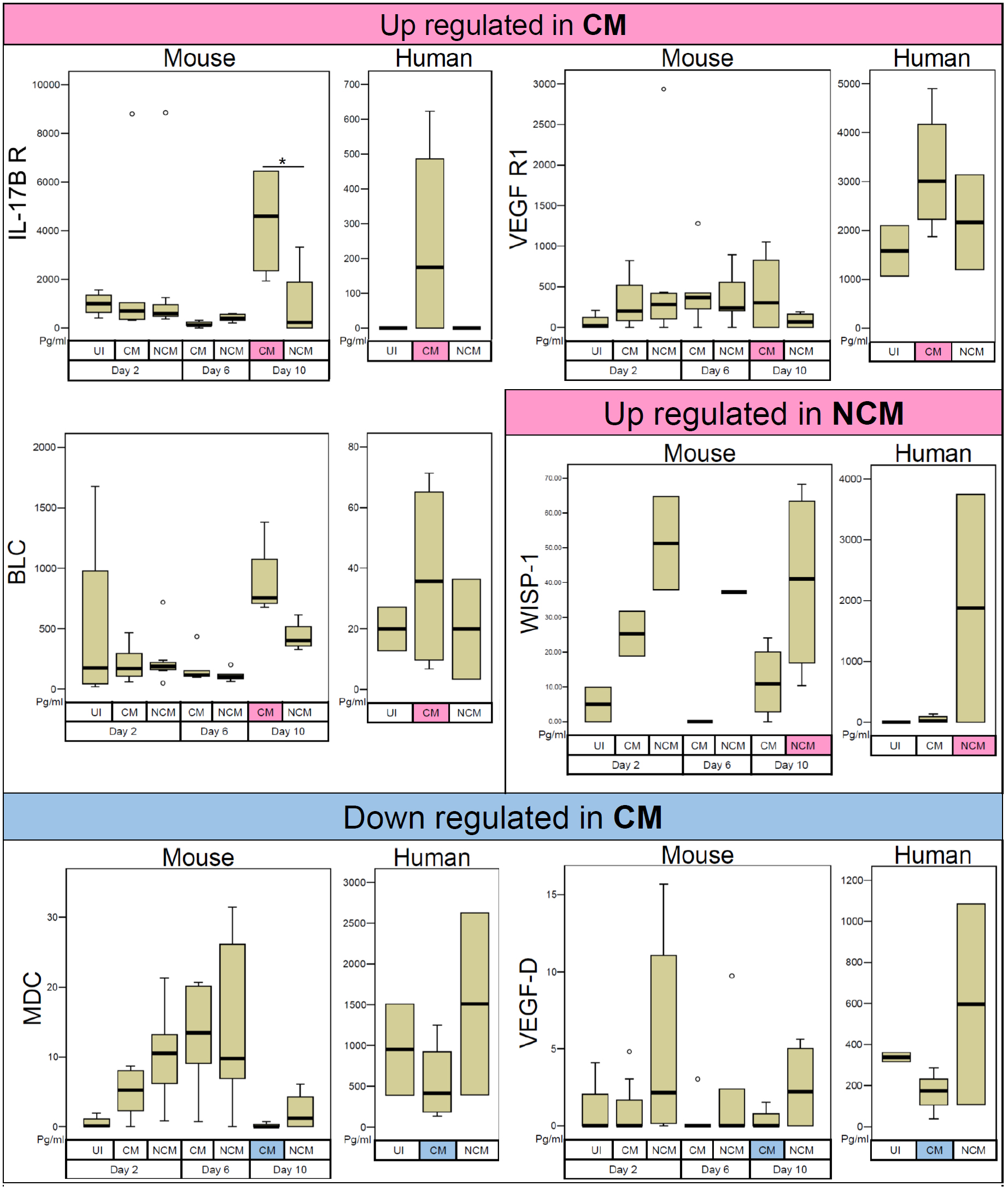

Supplement: FIG S7 [file mbio.03708-21-sf007.jpg]

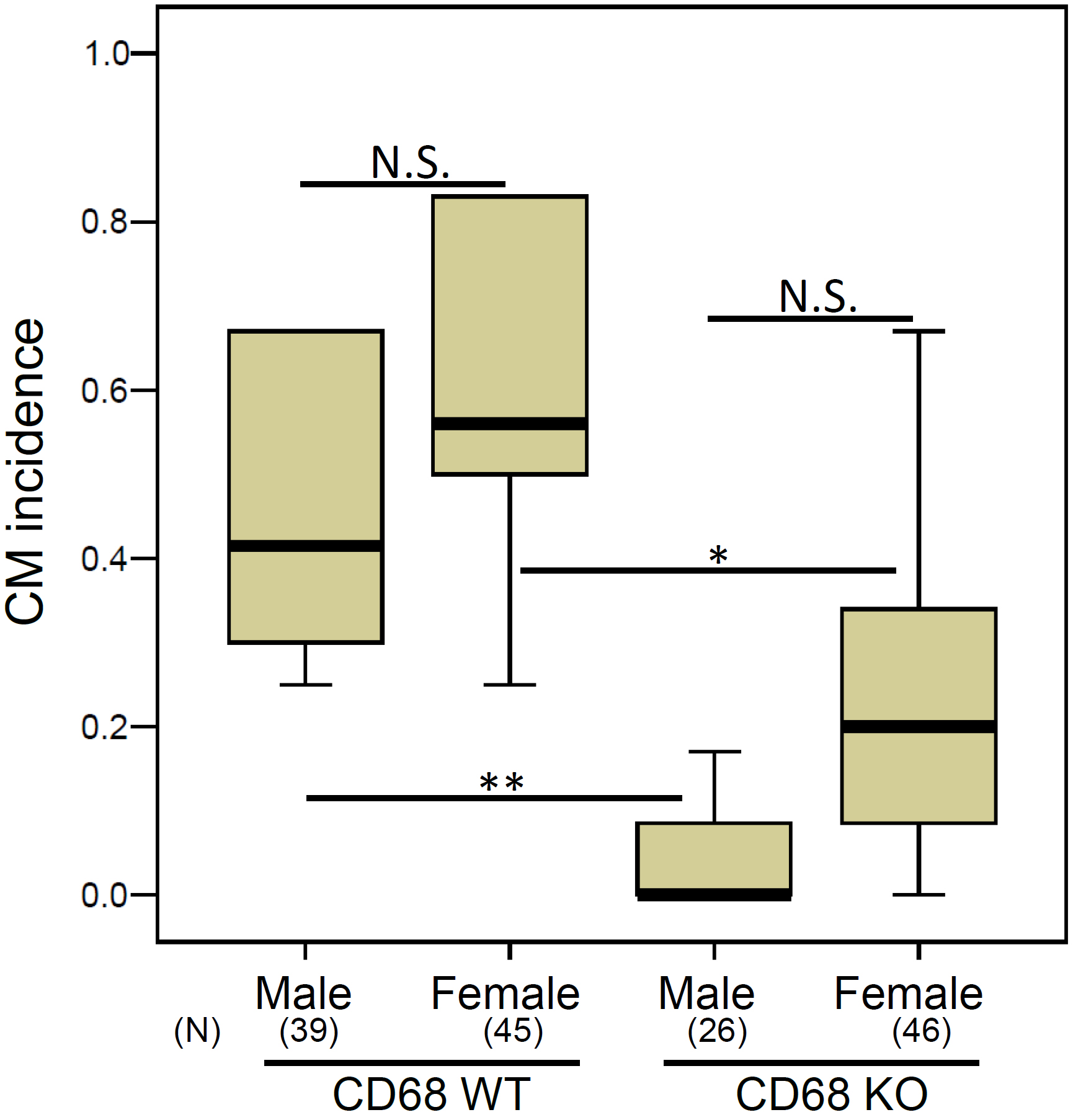

Supplement: FIG S8 [file mbio.03708-21-sf008.jpg]
